# Supplementary material for: Down-Regulation of AKT Proteins Slows the Growth of Mutant-KRAS Pancreatic Tumors
Source: Cells. 2024 Jun 19;13(12):1061. doi: 10.3390/cells13121061 (PMC11202146; doi:10.3390/cells13121061)
Supplement: Supplementary file 1 [file cells-13-01061-s001.zip › cells-3023843-supplementary.pdf]

SUPPLEMENTARY FIGURES

Down-regulation of AKT proteins slows the growth of mutant-KRAS pancreatic tumors

Chuankai Chen[1, 2], Ya-Ping Jiang[1], Inchul You[3], Nathanael S. Gray[3], and Richard Z. Lin[1.4]

[1]Department of Physiology & Biophysics, Stony Brook University, Stony Brook, New York, USA;

[2]Graduate Program in Genetics, Stony Brook University, New York, USA

[3] Department of Chemical and Systems Biology, ChEM-H, and Stanford Cancer Institute, Stanford School of Medicine, Stanford University, Stanford, California 94305, USA;

[4]Northport VA Medical Center, Northport, New York, USA

*Corresponding author: Richard Z. Lin, Stony Brook University, BST 6-180, Stony Brook NY 11790, Tel: 631-444-1638, Email: Richard.Lin@stonybrook.edu.

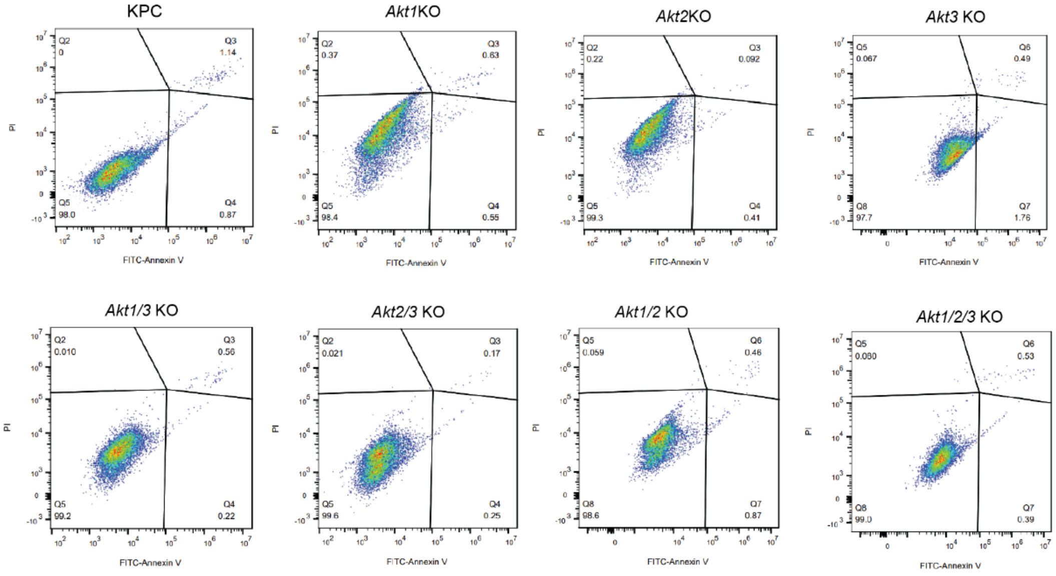

Supplemental Figure S1. KPC and *Akt1/2/3*KO cells showed comparable apoptotic rates.The apoptosis assay by ANNEXIN-V and PI staining and flow cytometry analysis. The cells were set up as the cell counting assay in Figure 3(A) and evaluated on day 4 by flow cytometry, representative images of technical replicates (n=2) are shown.

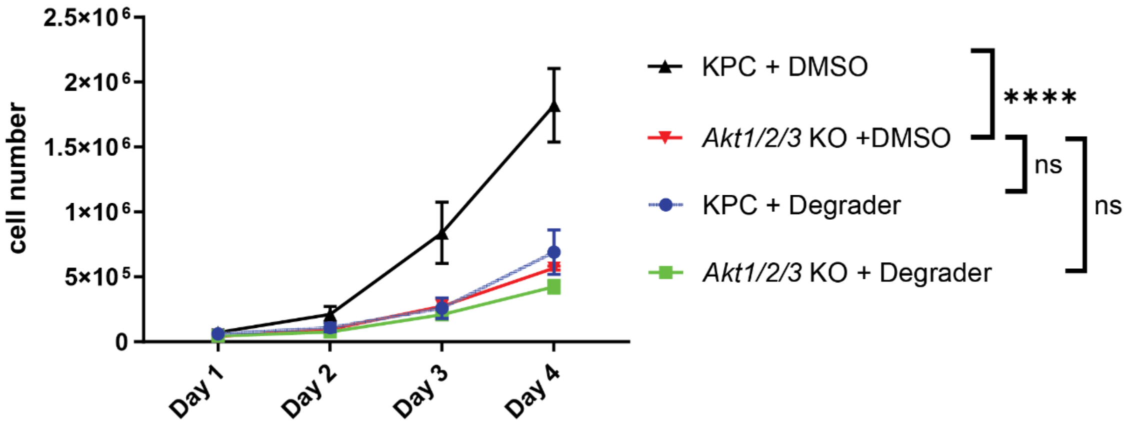

Supplemental Figure S2. AKT Degrader phenocopied *Akt1/2/3* genetic ablation in impeding cell growth. 50,000 cells were seeded in triplicates in each well of 6-well plates in 5% FBS DMEM with DMSO, or 500nM AKT Degrader. Cells were counted over the following 4 days. The experiment was repeated three times. Two-way ANOVA was performed, followed by uncorrected Fisher's LSD multiple comparisons of the indicated pairs. ****$p<0.0001$

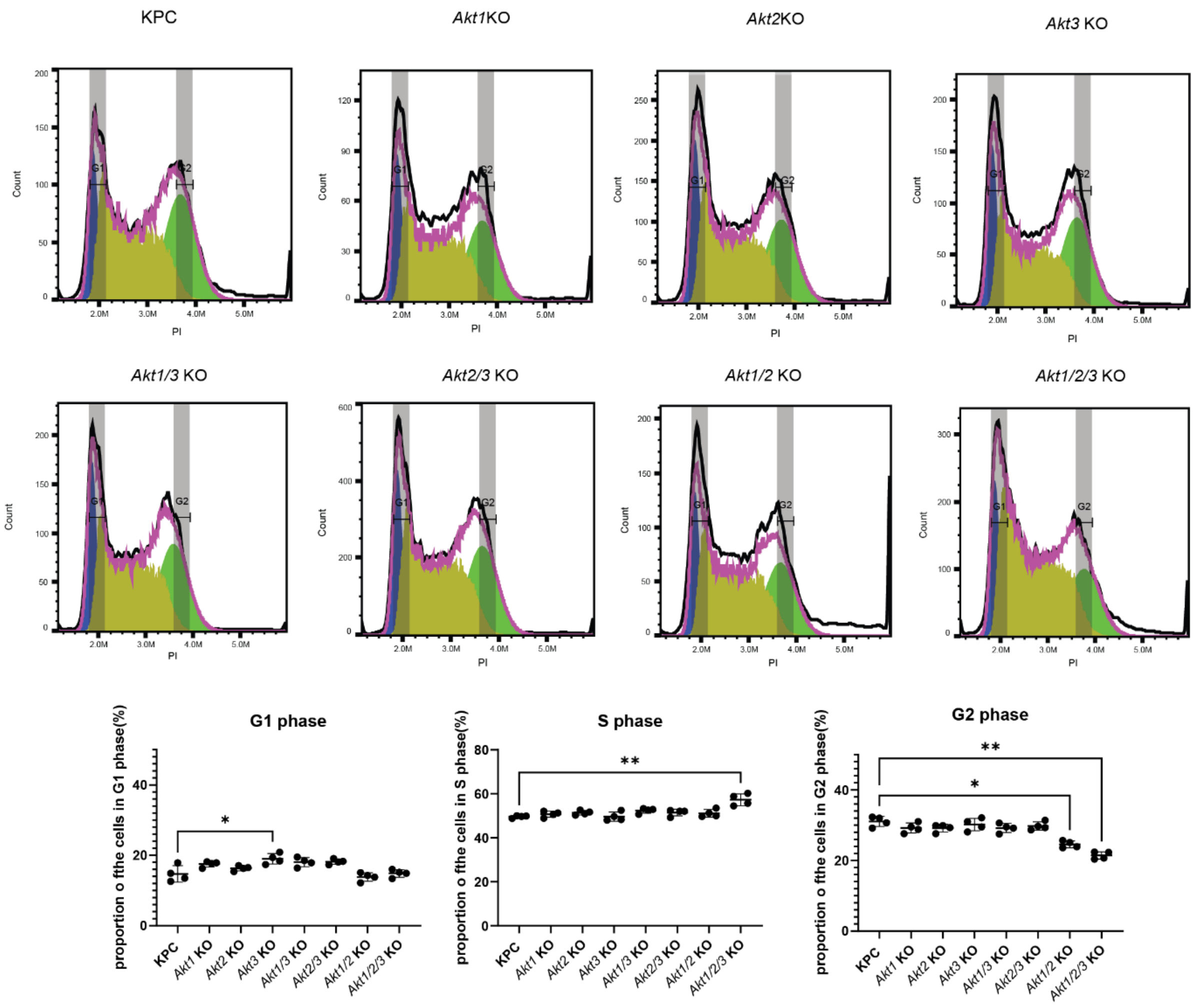

Supplemental Figure S3. KPC and *Akt1/2/3*KO cells showed comparable cell cycle distribution. Cell cycle analysis based on PI staining of the nuclear DNA. The cells were set up as in Figure 3(A) in technical replicates (n=4), and evaluated on day 2 by flow cytometry. Kruskal–Wallis ANOVA (nonparametric) tests were performed comparing proportions of G1 (P=0.0019), S (P=0.0112) and G2 (P=0.0051). Dunn's multiple comparisons test was made between KPC and each of the other lines, but only statistically significant comparisons are shown. * P < 0.05, ** P < 0.01, *** P < 0.001, **** P<0.0001.

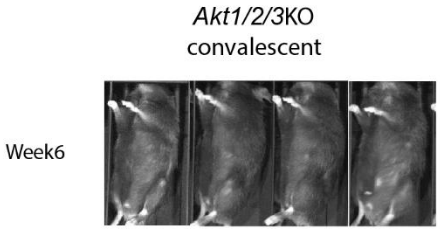

Supplemental Figure S4. *Akt1/2/3*KO cells-implanted mice had long-term survivors. Representative IVIS images of all four tumor-free long-term survivors implanted with *Akt1/2/3*KO cells at week 6 post-implantation.

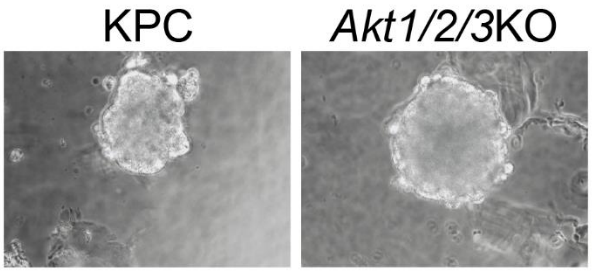

Supplemental Figure S5. Both KPC and *Akt1/2/3*KO cells were capable of anchorage-independent growth. 3D culture in 0.24% methylcellulose 10%FBS DMEM indicates that both KPC and *Akt1/2/3* KO cells are capable of anchorage-independent growth. Pictures were taken at 40X magnification with an inverse microscope.

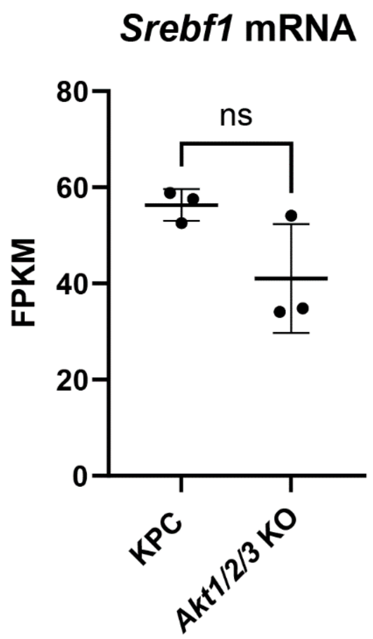

Supplemental Figure S6. The transcript level of *Srebf1* in KPC versus *Akt1/2/3*KO cells.

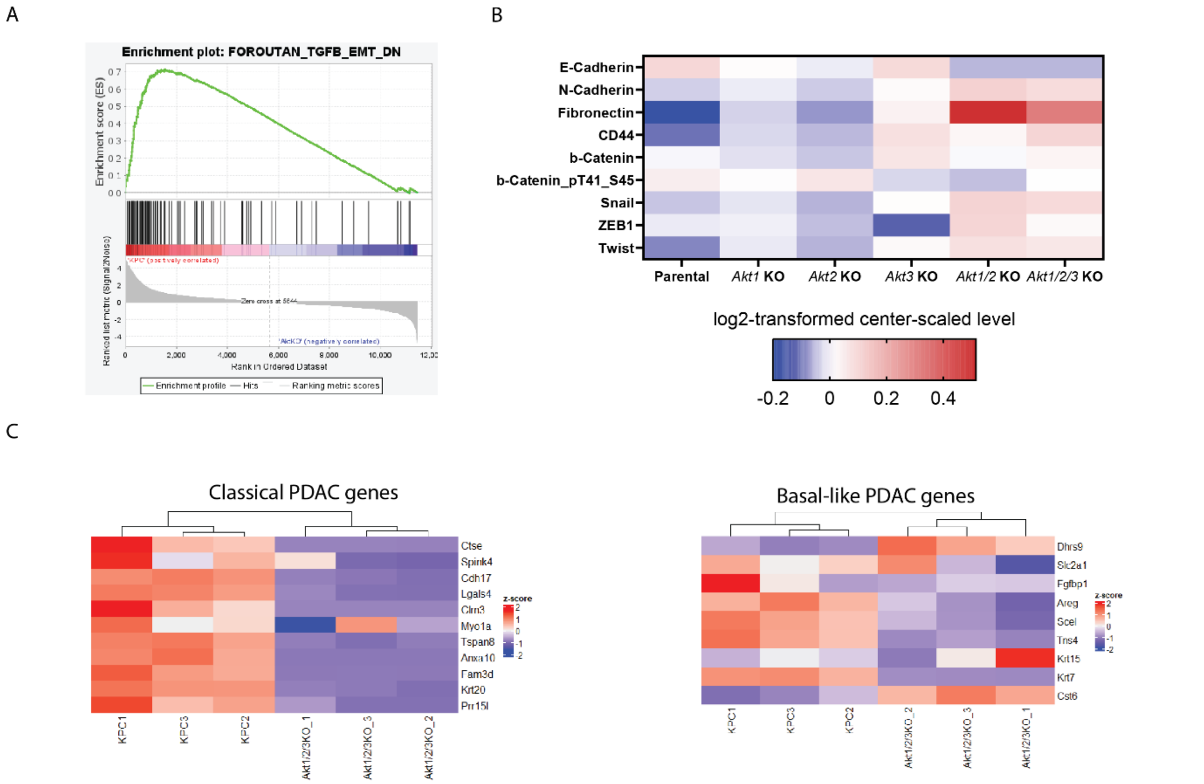

Supplemental Figure S7. RNA-seq and RPPA suggest *Akt/1/2/3*KO cells undergo epithelial-to-mesenchymal transition (EMT). (A) Representative EMT gene set in GSEA pathway enrichment analysis. (B) Relative protein levels by RPPA of EMT-signature genes of the indicated groups. (C) Heat maps comparing KPC and *Akt1/2/3*KO cells in terms of human PDAC subtypes signature genes.

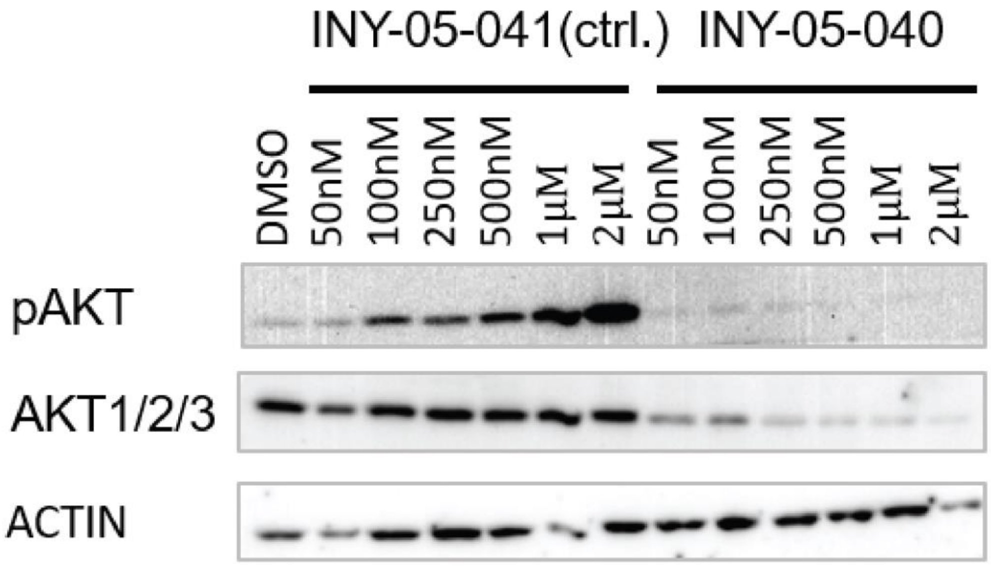

Supplemental Figure S8. Titration for the optimal working concentration of AKT degrader in KPC cells. Immunoblot for total AKT and phosphorylated AKT of KPC cells treated with increasing concentrations of AKT degrader INY-05-040 or the control compound INY-05-041.
